# UAV‐based imaging platform for monitoring maize growth throughout development

**DOI:** 10.1002/pld3.230

**Published:** 2020-06-08

**Authors:** Sara B. Tirado, Candice N. Hirsch, Nathan M. Springer

**Affiliations:** ^1^ Department of Agronomy and Plant Genetics University of Minnesota Saint Paul MN USA; ^2^ Department of Plant and Microbial Biology University of Minnesota Saint Paul MN USA

**Keywords:** maize, plant height, UAV phenotyping

## Abstract

Plant height (PH) data collected at high temporal resolutions can give insight into how genotype and environmental variation influence plant growth. However, in order to increase the temporal resolution of PH data collection, more robust, rapid, and low‐cost methods are needed to evaluate field plots than those currently available. Due to their low cost and high functionality, unmanned aerial vehicles (UAVs) provide an efficient means for collecting height at various stages throughout development. We have developed a procedure for utilizing structure from motion algorithms to collect PH from RGB drone imagery and have used this platform to characterize a yield trial consisting of 24 maize hybrids planted in replicate under two dates and three planting densities. PH data was collected using both weekly UAV flights and manual measurements. The comparisons of UAV‐based and manually acquired PH measurements revealed sources of error in measuring PH and were used to develop a robust pipeline for generating UAV‐based PH estimates. This pipeline was utilized to document differences in the rate of growth between genotypes and planting dates. Our results also demonstrate that growth rates generated by PH measurements collected at multiple timepoints early in development can be useful in improving predictions of PH at the end of the season. This method provides a low cost, high throughput method for evaluating plant growth in response to environmental stimuli on a plot basis that can be implemented at the scale of a breeding program.

## 1 INTRODUCTION

Plant height (PH) serves as a major growth indicator and can be used for assessing crop productivity and making crop management decisions. PH has been shown to be linked to nitrogen (N) nutrition during vegetative development in maize (Yon, Jaja, Mcclure, & Hayes, 2011; Freeman et al.,  2007), making it useful for assessing spatial variability in crop response to N. PH can, therefore, help guide precision agriculture practices such as variable‐rate N applications within the field. PH has also been linked to plant biomass in maize and barley crops (Bendig et al., 2014; Freeman et al., 2007; Li et al., 2016). Studies have found that PH measurements at early to mid‐developmental stages in maize are correlated to grain yield (Yin, Mcclure et al., 2011; Katsvairo, Cox, & Van Es, 2003), and can be useful for forecasting crop yields (Yin, Jaja, et al., 2011) or improving yield predictions (Sharma & Franzen, 2014). These findings suggest that tracking PH at these earlier stages and throughout development can be useful for identifying superior cultivars in plant breeding programs and for developing management practices that account for spatial heterogeneity in production fields. However, there is still substantial variation across these studies in the level of utility of early‐season PH in predicting end of season traits such as yield making this a topic that needs to be further evaluated. To maximize the utility of PH data, and to address some of the uncertainties regarding the utility of PH, data for large‐scale experiments at various stages throughout development needs to be gathered. With current methods for measuring height, this can be challenging. Similar to many phenotypic traits, current practices of gathering height in field settings involve physically measuring PH with a large ruler, which is time consuming and difficult to implement on a large scale. Ruler measurements are also subject to user bias and error, decreasing the accuracy and utility of these measurements.

Remote sensing technology has been proposed as an efficient means for collecting rapid and objective measurements for PH (Araus & Cairns, 2014; Madec et al., 2017; Yang et al., 2017). Unmanned aerial vehicles (UAVs) have proven to be a promising platform for collecting remote sensing data as they are inexpensive yet capable of achieving very high spatial and temporal resolutions. Many modern UAVs also have the functionality of flying automatically along a path specified by the user through mission planning software, increasing reproducibility through time. Commercial UAV platforms come equipped with RGB cameras that can be utilized to capture images along the field to create 3D field reconstructions using structure from motion (SfM) and multiview stereo (MVS) algorithms which rely on estimating the 3D structure of a scene utilizing a set of 2D images (James & Robson, 2012). The speed and ease with which UAVs can be flown for data acquisition provide advantages in scale and temporal resolution over other remote sensing methods utilized for creating 3D models, such as light detection and ranging (LiDAR), which involve high costs in data acquisition and processing (Díaz‐Varela, Rosa, León, & Zarco‐Tejada, 2015). However, there is substantial improvement that is needed in developing tools and approaches for simple implementation of UAV technology to generating phenotypic data in field settings.

There are examples of success in using RGB sensors to extract a number of traits that, if taken through time, can give us insight into plant growth and the relative effects of genetic and environmental variables on these traits. Canopy coverage estimates have been gathered for soybeans using digital cameras and these have been found to highly correlate to canopy light interception measurements (Purcell, 2000) and grain yield (Xavier, Hall, Hearst, Cherkauer, & Rainey, 2017). Previous studies have estimated PH from UAV imagery in sorghum (Chang, Jung, Maeda, & Landivar, 2017; Shi et al., 2016; Watanabe et al., 2017), wheat (Holman et al., 2016; Madec et al., 2017; Michalski et al., 2018), cotton (Feng, Sudduth, Vories, Zhang, & Zhou, 2018), barley (Bendig et al., 2014), and maize (Anderson II et al., 2019; Anthony, Elbaum, Lorenz, & Detweiler, 2014; Geipel, Link, and Claupein, 2014; Grenzdörffer, 2014; Su et al., 2019; Varela et al., 2017). Although these studies have reported high correlation in PH measurements for various dates pooled together to manual measurements, they lack estimates of how daily correlations of imagery‐derived PH (PH_UAV_) and ruler‐derived PH (PH_R_) vary throughout different growth stages and how this compares to the inherent error in PH_R_ measurements.

Taking into account past efforts in height estimation from UAV platforms, the present work aims to implement SfM algorithms for the estimation of PH_UAV_ for a maize field throughout development and compare these measurements to daily PH_R_ measurements collected using the traditional manual ruler‐based method. We tested the error in PH_R_ measurements and assessed the correlation of PH_UAV_ to PH_R_ measurements. We also evaluated applications in which this method could be used such as in estimating terminal height from early‐season PH_UAV_ data.

## 2 MATERIALS AND METHODS

### 2.1 Experimental design

Two replicates of 12 maize hybrids were planted in 4‐row plots at two dates (14 May 2018 and 21 May 2018) and three densities (60 k, 90 k, and 120 k plants per hectare) following a randomized complete block design blocked by planting date and replicate within planting date in St Paul, MN in the summer of 2018 (Figure S1). Each row was 15 ft long center‐to‐center with 12 ft of plots and 3 ft of alleys and 30 in spacing between rows. There were a total of 144 four‐row plots planted for this experiment. The 12 hybrid genotypes utilized were generated by crossing ex‐PVP lines selected due to their past use in production settings and the availability of seed. This experiment was grown on two acres. Nine 1 × 1 m ground targets were distributed around the border and internal alleys of the field for use as ground control points (GCPs) based on previously developed optimization algorithms (Gomez‐Candon, De Castro, & Granados, 2014).

### 2.2 Drone image data collection

**FIGURE 1:**
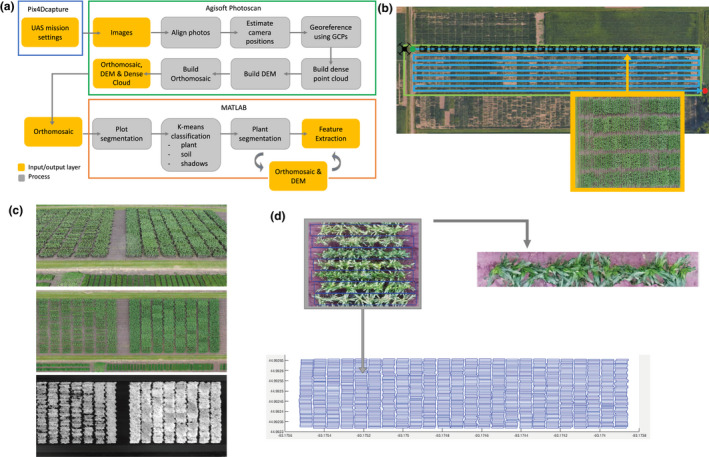
Procedure for feature extraction from UAV images. (a) Image generation and processing pipeline. (b) UAS flight mission structure for gathering images. (c) Dense cloud (top), orthomosaic (middle) and DEM (bottom) output from Agisoft Photoscan Professional Edition. (d) Plot boundary extraction from grid shapefile for plot segmentation. UAV, unmanned aerial vehicle

The field was imaged approximately weekly from planting until plants reached terminal height using a DJI Phantom 4 Advanced drone flown 30 m above ground to achieve a ground sampling distance (GSD), measured as the ground distance represented between the centers of two neighboring pixels, of approximately 0.82 cm. SfM algorithms for 3D reconstruction of field points from 2D images rely on finding correspondences between images. Wind can cause issues by causing the object to move between two consecutive recordings. This can be reduced by using a high measuring repetition rate (Paulus, 2019). Images were collected in a grid pattern using 85% frontlap and 85% sidelap to maximize reconstruction efficiency (Figure 1b). A total of 615 images were gathered per mission and a total of twelve missions were conducted throughout the season.

### 2.3 Manual measurement data collection

Manual measurements for PH (PH_R_(2)) were collected on the same day as eight of the drone flights by measuring two plants per row from each of the middle two rows of each four‐row plot using a ruler. PH_R_ was measured as the distance between the ground and the topmost freestanding vegetative part of the plant until plants reached reproductive growth, and then, to the top point of the tassel. For 18 selected plots, hand measurements of all plants in one of the middle two rows (PH_R_(All)) were collected to get estimates of variance in height within a row. The 18 plots comprised of one replicate of all three densities in the early planting date for six randomly selected genotypes (DK3IIH6 × LH198, LH198 × PHN46, LH82 × PHK76, PHB47 × LH198, PHB47 × PHP02, and PHK56 × PHK76).

### 2.4 Data processing workflow

Agisoft software (Agisoft Photoscan Professional Edition v1.4.4, 2018) was used to process the collected imagery and generate crop surface models (CSM) and RGB orthomosaics for each date following the developed workflow (Figure 1a). Processing steps implemented in Photoscan include feature matching, solving for camera intrinsic and extrinsic orientation parameters, building a dense point cloud, building a field orthomosaic, and building a digital elevation model as specified by Photoscan manual recommendations (Agisoft LLC, 2018; Figure 1c). Parameters set for image alignment included: high accuracy for obtaining camera position estimates, reference pair selection so that overlapping pairs of photos are selected based on measured camera locations, a key point limit of 40,000 points, and a tie point limit of 4,000 points. GCPs were then used to optimize camera alignment. For generating the dense point cloud, the quality value was set to High with Moderate depth filtering to optimize processing time and model quality.

**FIGURE 2:**
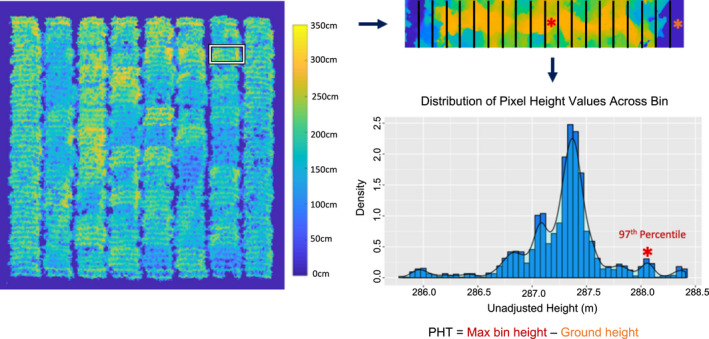
Method for extracting mean PH_UAV_ values for a given plot. The plot is segmented and broken down into 20 bins. The 3rd and 97th percentiles for each bin are extracted and used to calculate the average plot height by subtracting the minimum 3rd percentile of all bins from the 97th percentile of each bin

QGIS software (QGIS v2.18.9, 2017) was utilized for plot boundary extraction by overlaying a grid based on plot size and spacing and exporting plot boundary coordinates for the middle two rows of each plot (Figure 1d). Custom MATLAB algorithms were developed to process the CSMs and orthomosaics to extract height estimates for individual plots. PH was extracted by segmenting individual plots from the CSMs, overlaying a grid with 20 bins along the plot and extracting the 3rd percentile and 97th percentile height values for each bin. To select these optimal percentiles, the minimum 1st, 3rd, and 5th percentile values from all bins were first extracted and defined as ground height estimates for a subset of plots and the resulting plot height estimates compared to each other to select the optimal ground height extraction procedure. All three methods utilizing the different percentiles represented plot ground height values in the alleys; however, the 3rd percentile was selected as this value reduced the amount of error in ground height extraction compared to the 1st percentile due to outlier points present in the 3D models and the subsequent DEMs that were generated. This percentile also produced plot height estimates that had a higher correlation to ground truth measurements compared to utilizing the 5th percentile. The 97th percentile was selected following the same logic and to keep the procedure consistent. To extract the PH_UAV_ value for each bin, the ground height value (minimum 3rd percentile across bins) was subtracted from the 97th percentile height value in each bin. To calculate the mean PH_UAV_ per plot, a trimmed mean function was implemented on the PH_UAV_ values of the middle 12 bins of the plot trimming the two bins with the highest and lowest height values and determining the average of the remaining 10 height values (Figure 2). Only the middle 12 bins for the middle two rows in each plot were assessed when calculating an average PH value to prevent the average height for a given plot to be biased by the height of plants near alleys or different density treatments. A piecewise polynomial spline of order 20 was fit using the MATLAB splinefit function to better represent the continuous plant growth that occurs throughout the growing season (Lundgren, 2019). To assess how growth changes through time, we then extracted the first derivative, or slope, of the curve to get change in height through time.

### 2.5 Statistical analyses

A total of 1,152 mean plot PH_UAV_ values were extracted across eight timepoints. Outliers were identified for each timepoint as those values that were less than or larger than the mean PH_UAV_ value for that date plus or minus the standard deviation multiplied by a factor of 21/2. A total of 17 outlier points across all dates were identified and removed from the data set for subsequent analysis. It should be noted that while there was a strong wind event that caused substantial lodging in the experiment (see below), these outlier points were random and not enriched for data points collected at or shortly after the lodging event. To measure the strength of the linear relationship between different PH measurements, Pearson's correlation was utilized.

Variation in mean plot PH values was partitioned into genotype, planting date, density, genotype‐by‐density interaction, genotype‐by‐planting date interaction, and replicate all treated as fixed effects with the linear model $y_{\mathrm{ijkl}}=u+g_{i}+e_{j}+d_{k}+r{\left(e\right)}_{l\left(j\right)}+{\left(\mathrm{ge}\right)}_{i*j}+{\left(\mathrm{gd}\right)}_{i*k}+e_{\mathrm{ijkl}}$ where $y_{\mathrm{ijkl}}$ is the phenotype value of the *i*th genotype in the *j*th planting date in the *k*th planting density of the *l*th replication; u is the phenotypic mean across planting dates and planting densities; $g_{i}$ is the *i*th genotype effect; $e_{j}$ is the *j*th planting date effect, $d_{k}$ is the *k*th planting density effect; $r{\left(e\right)}_{l\left(j\right)}$ is the *l*th replication effect nested within the *j*th planting date; ${\left(\mathrm{ge}\right)}_{i*j}$ is the interaction effect of the *i*th genotype by the *j*th planting date; ${\left(\mathrm{gd}\right)}_{i*k}$ is the interaction effect of the *i*th genotype by the *k*th planting density; and $e_{\mathrm{ijkl}}$ is the residual effect. To test the significance of the various effect variables of the linear model on PH, we used the ANOVA function of the R stats package (R Core Team, 2012).

### 2.6 Model development for predicting terminal height

A piecewise polynomial function of 20 degrees was fit to PH_UAV_ values for each plot through time to create a smooth curve with optimal fit. The first derivative of the fitted curve was then extracted to get change in height through time (Figure 6b). The slope curve was then broken up into 100 equidistant timepoints for each plot. The contribution of each timepoint to the prediction of mean terminal height for each respective plot, based on hand measurements after plants had reached the reproductive stage, was tested utilizing a linear regression model. A new linear regression model was then created to predict terminal height utilizing slope values for the timepoints that provided the largest contribution, in this case more than 40%, of the observed variation for terminal hand height. The final model contained the terminal height as the response variable and the slope at nine timepoints as the predictor variables (Figure 6b). This model was derived from 1/4 of the data points and tested to predict the terminal height of the other 3/4 of the plots.

## 3 RESULTS AND DISCUSSION

Our goal was to implement a robust low‐cost platform for efficient estimation of PH for maize plots. High‐throughput collection of PH measurements throughout the growing season can provide a better understanding of how different environments influence maize growth and can help to dissect the underlying cause of end of season genotype × environment (G × E) interactions that are commonly observed. In order to develop a robust PH collection pipeline and to begin to consider the factors that influence plant growth rates, we planted a set of hybrids in a design that incorporated multiple planting densities and planting dates such that plants would be exposed to similar environments but at different growth stages (Figure S1). To decrease the impact of neighboring plots, each plot was grown as a four‐row plot and only plants in the middle two rows were measured. Manual measurements were taken using a ruler on the dates that aerial images were obtained. Analysis of variance showed significant variation in final PH within this experimental design due to genotype, planting date, and density (Table 1).

**TABLE 1:** Analysis of variance of hand measured plant height for 12 hybrids across 6 environments at physiological maturity (09 August 2018)

Variables	Degrees of Freedom	Sum of Squares	Pr(>|t|)
Genotype	11	40,518	<2e‐16***
Planting Date	1	31,241	<2e‐16***
Density	2	764	0.007114**
Replicate	1	146	0.161268
Genotype*Planting Date	11	7,194	1.19e‐10***
Genotype*Density	22	2042	0.214862
Planting Date*Density	2	1,418	0.000151***
Residuals	93	6,810	

Abbreviation: N.S., not significant.

* Significant at *p* = .05.

** Significant at *p* = .01.

*** Significant at *p* = .001.

Aerial images of the field were collected throughout the growing season until plants reached terminal height (approximately weekly, weather permitting). The images were used to create high resolution 3D models, field orthomosaics, and DEMs using existing SfM algorithms (Figure 1). There are multiple methods that have been utilized for extracting PH from UAV images. A common approach used is the difference based method (DBM), which involves subtracting a digital terrain model (DTM) of the bare ground from the DEM (Varela et al., 2017). The DTM can be generated by flying the bare ground but requires an additional day of data collection and processing and can introduce error due to minor fluctuations in flight altitude within and across dates that are common with most commercial drone platforms. Other approaches attempt to create the DTM by identifying and extracting ground points from the pointcloud and interpolating the height of intermediate points to create a surface model of the ground, and then, taking the difference between the DTM and DEM, but these approaches require processing large 3D point clouds and finding optimal parameters for the interpolation algorithm which can be computationally intensive (Su et al., 2019). Different algorithms for generating a DTM including DBM and three point cloud interpolation methods were evaluated by Anderson II et al. (2019) to see which provides higher accuracy when estimating plot heights. They found that all methods had similar, consistent performance in flat, uniform fields similar to those used in breeding trials.

Rather than generating a DTM, we developed a pipeline that involves identifying ground height values individually for each plot directly from the DEMs of a given date when calculating mean plot height. For each plot, the height was estimated from the DEMs derived from UAV imagery (PH_UAV_) by calculating the difference between the ground height and surface height of plant material across each plot and calculating a mean plot height value (Figure 2, see methods). It is important to note that this method relies upon determining ground height values based on visibility of ground pixels within the alleys separating adjacent plants. This means that this method requires spaces between plots that are free of weeds. The viability of this approach would be limited in situations of high weed pressure or nonexistent alleys, as would occur in production settings.

### 3.1 Error within ruler height measurements

A critical question for any application using PH_UAV_ is the quality of the height estimates that are obtained through this high throughput measurement. However, there are multiple complicating factors in truly assessing the quality of PH_UAV_ estimates. An important factor is that the manual measurements of PH (PH_R_(2)) that are used as the “ground truth” for determining the accuracy of PH_UAV_ estimates can include some level of error. In order to begin to understand the quality of the PH_R_ estimates, we replicated hand measurements of the same plants for a subset of plots at three of the timepoints (13 June 18, 17 July 18, and 9 August 18). To do this, we measured the same two plants per row twice for 12 randomly selected plots by having personnel return to the same plots when taking manual measurements and measuring the same plants which were tagged. An assessment of the correlation and error in replicated hand measurements of the individual plants and plot mean PH_R_(2) revealed variation in the correlations and root mean square errors (RMSEs) (Table S1). In the earliest timepoint, which corresponds to crop vegetative stages of V6 and V4 for the first and second planting respectively, the correlation between ruler measurement replicates showed the lowest correlation (adj. r‐square value of .45 for individual plant measurements and .64 for plot mean measurements calculated using the two plants from the middle two rows). This correlation improved with later developmental stages. This improvement could be due to larger differences in height later in development due to the genotypic variation present. Similarly, the NRMSE between ruler measurement replicates was highest in the first timepoint but was consistently lower at later timepoints. The correlation at terminal PH was high (adj. r‐square value of .9 for individual plant measurements and .64 for plot mean measurements) and had a low amount of error (NRMSE of 0.03 for individual plant measurements and 0.01 for plot mean measurements; Table S1).

Another potential difference between PH_R_ and PH_UAV_ estimates of plot height is that manual measurements are often collected for a subset of plants in a row while the UAV measurements are often generated as a per plot mean height that samples a larger number of plants. To investigate the accuracy in plot mean PH_R_(2) obtained by measuring only two random plants per row, we randomly sampled two individual plant PH_R_ measurements at each timepoint twice for the 17 plots, in which all plants for one of the middle two rows were measured and calculated new replicates for plot mean PH_R_(2) measurements with the intention of assessing the accuracy and error in traditional methods of plot mean calculation. We replicated this procedure 100 times for each plot and timepoint to get an estimate of the average amount of error in PH_R_(2) mean values calculated by measuring only two plants per row. Plot mean PH_R_(2) measurements obtained from different sets of plants within a row showed a much lower correlation and larger error across most timepoints compared to the correlations between PH_R_ replicated measurements of the same two plants (Figure 4c; Table S1). These correlations are comparable to those obtained when correlating plot mean PH_UAV_ measurements to plot mean PH_R_(2) measurements (Figure 4a). This indicates that PH_R_ measurements can have substantial variation that is impacted by the number of plants measured and the selection of plants to be measured.

### 3.2 Correlation of ruler height to UAV‐derived height measurements

**FIGURE 3:**
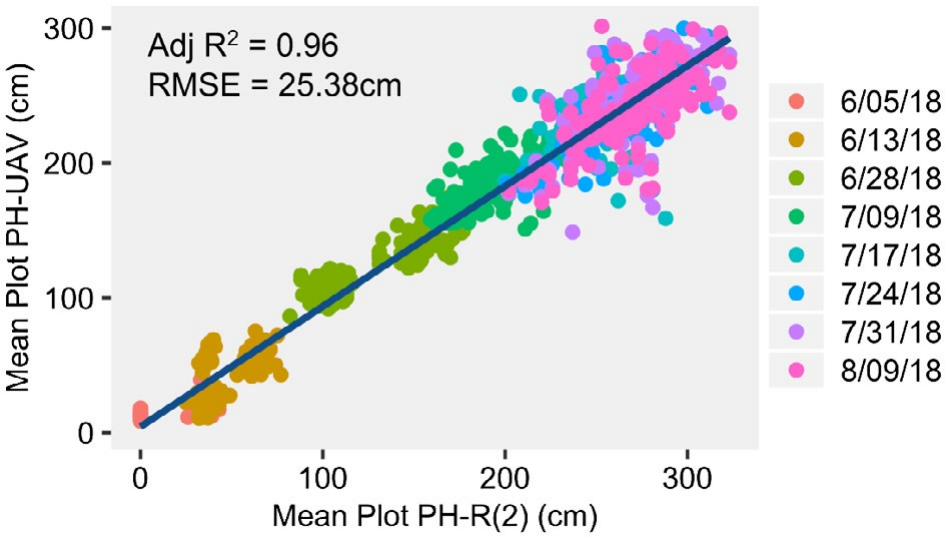
Pearson correlation of mean plot PH_UAV_ and mean plot PH_R_(2) across all dates

**FIGURE 4:**
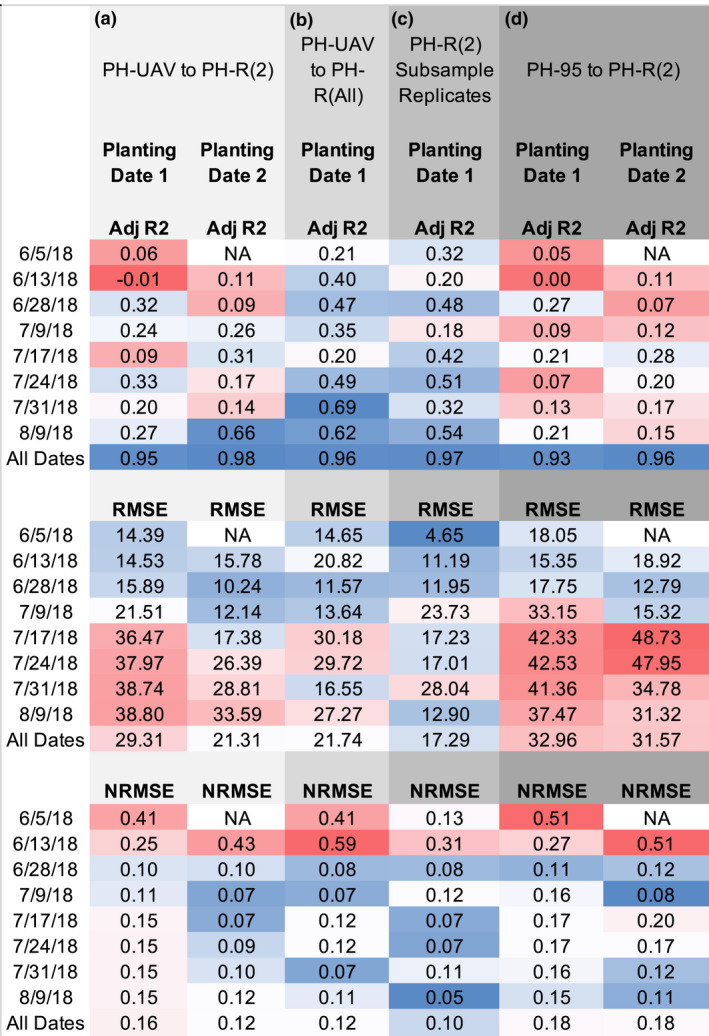
Adjusted r‐square values, root mean square error, and normalized root mean square error by mean for the linear correlation of various PH measurements. (a) UAV‐derived (PH_UAV_) plot mean height values compared to the respective hand‐measured plot mean height (PH_R_(2)) for plots where two plants per row were hand measured. (b) UAV‐derived (PH_UAV_) plot mean height values compared to the respective hand‐measured plot mean height (PH_R_(All)) for plots where all plants for one of the middle two rows were hand measured. (c) Means derived from two iterations of random sampling of hand‐measured height values for two plants across plots were all plants for one of the middle two rows were hand measured. (d) 95th percentile of height values from entire plot compared the respective hand‐measured plot mean height values (PH_R_(2)). PH, plant height; UAV, unmanned aerial vehicle

There are limitations in accuracy of ruler‐estimated heights for specific plants as well as for estimating the height of a plot. However, these measurements can still be used to assess the quality of PH_UAV_ measurements, if we accept that the quality of the correlations between PH_UAV_ and PH_R_ likely cannot be improved beyond the inherent errors in PH_R_ measurements. Compared to our manual height estimates, analysis of variance showed significant variation in terminal PH_UAV_ within this experimental design due to genotype and planting date; however, density had a smaller effect (Table S2). Comparison of PH_R_(2) and PH_UAV_ showed the overall correlation across all data collection dates was strong (adj. r‐square value of .96 for our dataset; Figure 3a), as has been previously shown in other studies (Varela et al., 2017). However, assessing the accuracy of all timepoints together from a biological standpoint offers little value. A more biologically relevant comparison is evaluating the correlations within individual timepoints of data collection. The correlations within a single date were much lower (Figure 4a). This is likely due to the fact that the level of variation for height on a single date tends to be relatively low. The RMSE for mean plot PH_UAV_ compared to the mean plot PH_R_(2) across all dates and both planting dates was 25.38 cm, but varied from 14.39 to 38.80 cm across dates for the first planting date and from 10.24 to 33.59 cm across dates for the second planting date (Figure 4a). The RMSE normalized by the mean plot PH_R_(2) measurements (NRMSE), on the contrary, showed that overall the error was higher at early developmental stages and then lowered and leveled off across later timepoints in both planting dates. The lower correlations and higher error for the first planting date were likely due to a storm that occurred on July 1st that produced strong winds. This storm caused large degrees of lodging, especially within plots in the early planting date treatment causing larger variability in height among plants within plots.

If we compare the error in PH_UAV_ measurements to that in PH_R_(2) replicated measurements for the subset of plots where two individual plants were hand‐measured twice, we see that the correlations of PH_R_(2) and PH_UAV_ measurements for the same plots increased throughout development, but were lower than the correlation between PH_R_(2) replicated measurements (Table S1). By terminal height, the correlation between PH_R_(2) and PH_UAV_ measurements was high (adj. r‐square value of .72) but had a substantial amount of error (Figure 3c). Unlike the high correlation and low RMSE obtained from plot mean PH_R_(2) measurement replicates calculated by measuring the same two plants per row, plot mean PH_R_(2) measurements obtained by sampling different sets of plants within a row showed a much lower correlation and larger error across most timepoints (Figure 4c; Table S1). These correlations between plot mean PH_R_(2) calculated by measuring varying plants in a row, however, are comparable to those obtained when correlating plot mean PH_UAV_ measurements to plot mean PH_R_(2) measurements (Figure 4a–c). The direct comparison of the PH_R_(2) and PH_UAV_ measurements obtained, therefore, yielded an error comparable to the inherent error in estimating PH_R_(2) across most timepoints.

Given that mean plot PH_UAV_ estimates take into account variation in height throughout a plot, we can better assess their correlations to mean plot PH_R_ measurements by looking at the subset of plots, where all plants in a row were measured. Doing so, we see that the correlations and error estimates of mean plot PH_UAV_ to mean plot PH_R_(All) greatly improve relative to those calculated by comparing mean plot PH_UAV_ estimates to mean plot PH_R_(2) measurements (Figure 4b. This indicates that mean plot PH_UAV_ measurements better resemble the true plot mean compared to the mean calculated from measurements of just a subset of plants per row.

It is worth noting that in our approach mean plot height values are obtained using a binning procedure across the plot (see methods), whereas most current methods for calculating mean plot height from UAV imagery extract a constant value, such as the 95th percentile, from the entire plot. However, the height for maize plants throughout a row can vary and extracting a single value might not capture this variation. When performing ruler measurements, researchers typically measure plants toward the center of a row due to the border effects that might cause plants near alleys to vary in height due to lower competition for sunlight, water, and nutrients. Thus, we decided to collect height across bins throughout the length of the plot and only utilize height values for bins toward the center of the plot to calculate an average plot height. To assess whether our binning approach provided a more informative framework for assessing the average height of an entire plot as would be collected through traditional hand measurements, we extracted the 95th percentile height value (PH_95_) from entire plots without binning, subtracted the 3rd percentile ground height values for each plot same as in our binning approach, and compared their correlations to PH_R_(2) measurements. PH_95_ showed low correlation to PH_R_(2) and high error rate throughout most individual timepoints with an RMSE ranging from 15.32 to 48.73 cm (Figure 4d). These lower correlations and higher error estimates compared to the PH_UAV_ measurements obtained through our approach show the value in binning and extracting an average to represent height for plots, where height variation is present rather than extracting a single value.

### 3.3 UAV‐estimated heights can detect biological variation

**FIGURE 5:**
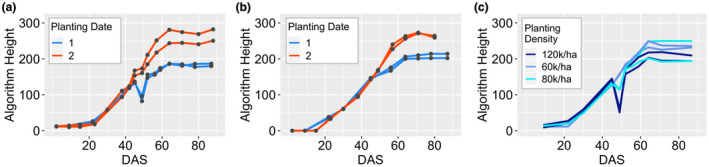
Height through time for various genotypes and treatments. (a and b) Height through time as measured by the UAV (a) and hand measurements (b) for two replicate plots of a single genotype (LH82 × DK3IIH6) planted under high density across two planting dates. Dots indicate timepoints of measurement. (c) Height through time as measured by the UAV for two replicate plots of a single genotype (LH82 × PHK76) in the early planting date treatment and the three planting densities. UAV, unmanned aerial vehicle

Our goal was to develop a low cost, high‐throughput platform for monitoring PH through time that would allow us to look at how genotypes develop and respond to different environmental conditions. A storm with high wind speeds occurred on July 1st of the 2018 growing season in St. Paul, providing a prime opportunity to evaluate the utility of this method to track plant responsiveness to environmental conditions. An example of this responsiveness can be seen in the height profiles for LH82 x DK3IIH6 plots, where plants at high density in the earlier planting date showed a reduction in height 45 days after sowing (Figure 5a). Normally, a reduction in height would be unexpected and may reveal issues with the height estimation platform, but this height reduction was due to the lodging that occurred as a result of the storm. We were not able to track the lodging event in the PH_R_ measurements (Figure 5b), as these measurements were not taken until several days after the wind storm and most plots recovered from the lodging prior to this measurement. UAV imagery provides an opportunity to collect data rapidly in response to events such as this storm event and track plants at regular intervals that is much more difficult to do with hand measurements.

We expected to note differences in height within genotypes and planting dates based on planting density. Plants grown at higher density tend to grow faster and taller when water and nutrient resources are not limited as they are competing with more plants for sunlight (Tetio‐Kagho & Gardner, 1988). Planting density did contribute to differences observed for lodging across genotypes in the early planting date. Plots planted at higher densities experienced a more severe degree of lodging when exposed to strong winds compared to plots planted at a lower density (Figure 5c). However, our data suggested that the realized height remained pretty stable across densities and that in many cases, the differences in height due to genotypic variation were reproducible across planting dates and densities (Figure S2a‐c).

The PH_UAV_ estimates were also able to document differences in growth rates throughout the growing season between genotypes. For example, LH82 × LH145 planted at high density in the late planting had a faster rate of growth compared to LH82 × DK3IIH6 planted in the same conditions as soon as 20 days after sowing (Figure S2c). LH82 × LH145 was able to maintain an advantage in terms of height throughout development and achieve a higher realized height at the end of the season. The ability to track these differences in growth patterns among genotypes that may or may not impact the end of season heights that are typically collected provides additional information to breeders in the process of selecting superior parents and hybrid combinations. Overall, visualization of the PH profiles for each genotype × condition suggested a fairly robust ability to monitor biological variation for height and responsiveness to environmental conditions under different growth conditions.

### 3.4 Predicting terminal height from height collected at earlier time points

**FIGURE 6:**
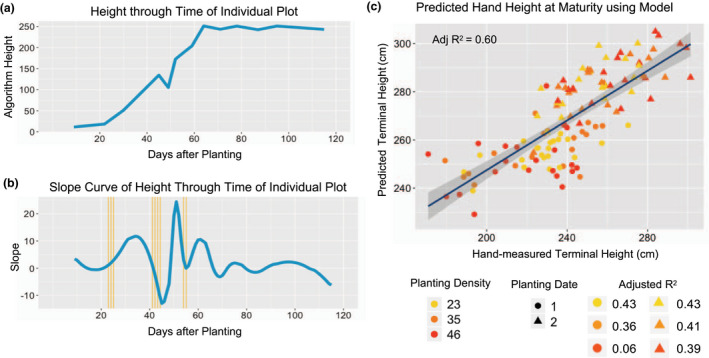
Predicting terminal height using rate of change in height at certain intervals. (a) Height through time for an individual plot as recorded by the UAV. (b) First derivative of the height through time spline curve in blue and the selected timepoints were the slope contributed most to the prediction of terminal height in yellow. (c) Correlation of predicted terminal height on the test dataset utilizing a linear regression model derived from the training dataset based on slope values of selected timepoints and the hand‐measured height values at maturity. UAV, unmanned aerial vehicle

Being able to estimate height for any given date is useful for tracking growth; however, being able to predict terminal height using height from earlier time points can allow for the assessment of end‐season performance at a time when you can still implement management practices to enhance it. This also can allow breeders to make selections of top‐performing plants that meet the desired phenotypic characteristics before making crosses thereby helping maximize their selection efficiency. We wanted to test whether height at early developmental stages could be predictive of terminal height. A previous study by Li et al. (2018) showed low correlations between PH collected for hybrid varieties at early developmental stages to their respective end of season height likely due to differences in the physiological basis for PH at both stages. Including temporal data for height, or rather the rate of change in height through time, could be more informative when predicting terminal height as it captures how different genotypes interact with the environment and how this interaction impacts the trait at hand. Therefore, rather than just using height at specific timepoints (Figure 6a) to predict terminal height, we wanted to utilize rate of growth at intervals in time. To do this, a B‐spline, or piecewise polynomial function, of 20 degrees was fit to create a smooth curve representative of true growth. Timepoints that showed a high contribution to terminal height were used to create a linear regression model to predict terminal height (see methods). The model was derived from 1/4 of the data points and tested to predict the other 3/4 of the plots. It contained nine timepoints, three of which were in the V2–V4 developmental stages, four in the V5–V7 stages and two in the V8–V10 stages. These timepoints all had biological significance as they fell upon areas of large inflection in the plant growth curve where a weather event or developmental time point caused a dramatic change in height. The first three timepoints which fall upon the V2–V4 developmental stage exhibited a large increase in height characteristic of early plant growth. The second set of three timepoints follow the storm that occurred around the V5–V7 developmental stage which caused a large degree of lodging just prior to their recovery. The last set of timepoints in the V8–V10 stages show a decline in rate of growth followed by a sudden increase. This decrease could be a consequence of weather variables such as temperature and moisture availability, whereas the increase could be due to the emergence of tassels. These timepoints were crucial points in plant development where plant growth responded to certain conditions that in turn affected the terminal height obtained.

This model had a high ability to predict terminal mean plot PH_UAV_ (adjusted r‐squared value of .63; Figure S3) and mean plot PH_R_(2) (adjusted r‐squared value of .60; Figure 6c). When looking at the different treatments separately, we can see that the ability to predict height across planting dates increases for the late planting treatment and the magnitude of this difference increases with higher planting densities. This is likely due to the challenges in estimating mean plot height for plots that suffered lodging and the higher density treatments in the early planting date experienced the most lodging early in the season. These height prediction results, although specific to this experiment, show the significant correlation that change in height from early season timepoints can have with terminal height and highlight the potential for developing tools to predict terminal height utilizing temporal PH measurements early in development. Maize plants uptake negligible amounts of nutrients such as nitrogen for the first weeks after emergence and then start to exponentially take up more nitrogen until the tasseling stage (Sharma & Bali, 2017). Being able to use height information gathered before the V10 developmental stage to predict terminal height and potentially stress can be a useful tool for guiding nutrient management practices early in development when plants are efficiently absorbing nutrients and can have a high response to fertilization.

## 4 CONCLUSIONS

High‐throughput collection of PH measurements throughout the growing season can allow a better understanding of how different environments influence maize growth and can reveal important factors affecting crop productivity. We have developed a novel approach for extracting plot PH_UAV_ from individual plots that involves estimating the ground height individually for each plot and using that to calculate mean plot height. Overall, PH_R_(2) and PH_UAV_ measurements showed a high correlation (adj. r‐square value of .96); however, correlations within PH estimates collected on a single date are much lower as they are impacted by the low variability in height expressed on a single date. The reproducibility and quality of PH_R_(2) estimates were found to vary throughout development with lower correlations and higher error rates observed in early vegetative stages, and improved correlations and lower error rates observed in later developmental stages. When assessing the error in generalizing mean PH_R_ for a plot by measuring only a subset of plants in a row, PH_R_ measurements were found to be greatly variable and impacted by the number and selection of plants sampled. Plot mean PH_R_(2) measurements obtained from different sets of plants within a row show a correlation and error across most timepoints comparable to those obtained when correlating plot mean PH_UAV_ measurements to plot mean PH_R_(2) measurements.

Implementing this pipeline to visualize height across different genotypes, planting densities, and planting date treatments showed a strong ability to monitor biological variation for height across development in response to the environment. Differences in rate of growth between genotypes and planting dates as well as in lodging responses between planting densities were documented. Moreover, we saw that growth rates generated by PH measurements collected at multiple timepoints early in development can be useful in improving predictions of PH at the end of the season. Together, these findings show the utility of using UAVs for collecting PH data at high temporal resolution to track differences in growth and to predict an end‐season trait early in development.

## CONFLICT OF INTEREST

The authors do not have any conflict of interest to declare.

## AUTHOR CONTRIBUTIONS

SBT, CNH, and NMS conceived the experiments; SBT conducted the experiments and analyses; SBT, CNH, and NMS wrote the manuscript.

## Supporting information

Data S1

Table S1‐S2

## Data Availability

The scripts and processes used to perform the image analyses and trait extraction are available at https://github.com/SBTirado/UAV_PH.git.

## References

[pld3230-bib-0001] AgiSoft PhotoScan Professional Edition (Version 1.4.4) (Software). (2018). Retrieved from http://www.agisoft.com/downloads/installer/

[pld3230-bib-0002] Agisoft LLC . (2018). Agisoft photoscan user manual: Professional Edition v.1.2.6. St. Petersburg, Russia: Agisoft LLC.

[pld3230-bib-0003] Anderson, S. L. II , Murray, S. C. , Malambo, L. , Ratcliff, C. , Popescu, S. C. , Cope, D. A. , … Thomasson, J. (2019). Prediction of maize grain yield before maturity using improved temporal height estimates of unmanned aerial systems. The Plant Phenome Journal, 2, 190004 10.2135/tppj2019.02.0004

[pld3230-bib-0004] Anthony, D. J. , Elbaum, S. G. , Lorenz, A. , & Detweiler, C. (2014). On crop height estimation with UAVs. IEEE/RSJ International Conference on Intelligent Robots and Systems, 2014, 4805–4812.

[pld3230-bib-0005] Araus, J. L. , & Cairns, J. E. (2014). Field high‐throughput phenotyping: The new crop breeding frontier. Trends in Plant Science, 19(1), 1360–1385. 10.1016/j.tplants.2013.09.008 24139902

[pld3230-bib-0007] Bendig, J. , Bolten, A. , Bennertz, S. , Broscheit, J. , Eichfuss, S. , & Bareth, G. (2014). Estimating biomass of barley using crop surface models (CSMs) derived from UAV‐based RGB imaging. Remote Sensing, 6(11), 10395–10412. 10.3390/rs61110395

[pld3230-bib-0008] Chang, A. , Jung, J. , Maeda, M. , & Landivar, J. (2017). Crop height monitoring with digital imagery from Unmanned Aerial System (UAS). Computers and Electronics in Agriculture, 141, 232–237. 10.1016/j.compag.2017.07.008

[pld3230-bib-0009] Díaz‐Varela, R. A. , Rosa, R. D. , León, L. , & Zarco‐Tejada, P. J. (2015). High‐resolution airborne UAV imagery to assess olive tree crown parameters using 3D photo reconstruction: Application in breeding trials. Remote Sensing, 7, 4213–4232. 10.3390/rs70404213

[pld3230-bib-0010] Feng, A. , Sudduth, K. A. , Vories, E. D. , Zhang, M. , & Zhou, J. (2018).Cotton Yield Estimation based on Plant Height From UAV‐based Imagery Data. *2018 ASABE Annual International Meeting* 10.13031/aim.201800483

[pld3230-bib-0011] Freeman, K. W. , Girma, K. , Arnall, D. B. , Mullen, R. W. , Martin, K. L. , Teal, R. K. , & Raun, W. R. (2007). By‐plant prediction of corn forage biomass and nitrogen uptake at various growth stages using remote sensing and plant height. Agronomy Journal, 99, 530–536. 10.2134/agronj2006.0135

[pld3230-bib-0012] Geipel, J. , Link, J. , & Claupein, W. (2014). Combined spectral and spatial modeling of corn yield based on aerial images and crop surface models acquired with an unmanned aircraft system. Remote Sensing, 6, 10335–10355. 10.3390/rs61110335

[pld3230-bib-0013] Gomez‐Candon, D. , De Castro, A. I. , & Lopez‐Granados, F. (2014). Assessing the accuracy of mosaics from unmanned aerial vehicle (UAV) imagery for precision agriculture purposes in wheat. Precision Agriculture, 15, 44–56. 10.1007/s11119-013-9335-4

[pld3230-bib-0014] Grenzdörffer, G. (2014). Crop height determination with UAS point clouds. ISPRS ‐ International Archives of the Photogrammetry, Remote Sensing and Spatial Information Sciences, XL‐1, 135–140. 10.5194/isprsarchives-XL-1-135-2014

[pld3230-bib-0015] Holman, F. H. , Riche, A. B. , Michalski, A. , Castle, M. , Wooster, M. J. , & Hawkesford, M. J. (2016). High throughput field phenotyping of wheat plant height and growth rate in field plot trials using UAV based remote sensing. Remote Sensing, 8, 1031 10.3390/rs8121031

[pld3230-bib-0016] James, M. , & Robson, S. (2012). Straightforward reconstruction of 3D surfaces and topography with a camera: Accuracy and geoscience application. Journal of Geophysical Research Atmospheres, 117(F3), F03017. 10.1029/2011JF002289

[pld3230-bib-0017] Katsvairo, T. W. , Cox, W. J. , Es, H. M. , & Glos, M. A. (2003). Spatial yield response of two corn hybrids at two nitrogen levels. Agronomy Journal, 95(4), 1012–1022. 10.2134/agronj2003.1012

[pld3230-bib-0018] Li, W. , Niu, Z. , Chen, H. , Li, D. , Wu, M. , & Zhao, W. (2016). Remote estimation of canopy height and aboveground biomass of maize using high‐resolution stereo images from a low‐cost unmanned aerial vehicle system. Ecological Indicators, 67, 637–648. 10.1016/j.ecolind.2016.03.036

[pld3230-bib-0019] Li, Z. , Coffey, L. , Garfin, J. , Miller, N. , White, M. , Spalding, E. , … Hirsch, C. (2018). Genotype‐by‐envrionment interactions affecting heterosis in maize. PLoSOne., 13(1), e0191321. 10.1371/journal.pone.0191321 PMC5771596 29342221

[pld3230-bib-0020] Lundgren, J. (2019). SPLINEFIT. Retrieved from https://www.mathworks.com/matlabcentral/fileexchange/71225‐splinefit.MATLAB Central File Exchange.

[pld3230-bib-0021] Madec, S. , Baret, F. , de Solan, B. , Thoas, S. , Dutartre, D. , Jezequel, S. , … Comar, A. (2017). High‐throughput phenotyping of plant height: comparing unmanned aerial vehicles and ground LiDAR estimates. Frontiers in Plant Science, 8, 2002. 10.3389/fpls.2017.02002 29230229 PMC5711830

[pld3230-bib-0022] Michalski, A. , Riche, A. , Castle, M. , Holman, F. , Hawkesford, M. , & Wooster, M. (2018).UAS in 3D Crop Modeling for Agricultural Research Bandrova T., & Konečný M. (Eds.), 2018 7th International Conference on Cartography and GIS, 1314–0604.

[pld3230-bib-0023] Paulus, S. (2019). Measuring crops in 3D: Using geometry for plant phenotyping. Plant Methods, 15, 103 10.1186/s13007-019-0490-0 31497064 PMC6719375

[pld3230-bib-0024] Purcell, L. C. (2000). Soybean canopy coverage and light interception measurements using digital imagery. Crop Science, 40, 834–837.

[pld3230-bib-0025] QGIS Development Team. (Version v2.18.9 ) (Software) .(2017). QGIS Geographic Information System. Open Source Geospatial Foundation Project. Retrieved from http://qgis.osgeo.org

[pld3230-bib-0026] R Core Team . (2012). R: A language and environment for statistical computing. Vienna, Austria: R Foundation for Statistical Computing Retrieved from http://www.R‐project.org/. ISBN 3‐900051‐07‐0.

[pld3230-bib-0027] Sharma, L. K. , & Bali, S. K. (2017). A review of methods to improve nitrogen use efficiency in agriculture. Sustainability, 10, 51 10.3390/su10010051

[pld3230-bib-0028] Sharma, L. K. , & Franzen, D. W. (2014). Use of corn height to improve the relationship between active optical sensor readings and yield estimates. Precision Agriculture, 15, 331–345. 10.1007/s11119-013-9330-9

[pld3230-bib-0029] Shi, Y. , Murray, S. C. , Rooney, W. L. , Valasek, J. , Olsenholler, J. A. , Pugh, N. A. , … Thomasson, J. A. (2016). Corn and sorghum phenotyping using a fixed‐wing UAV‐based remote sensing system.*SPIE Commercial + Scientific Sensing and Imaging* 10.1117/12.2228737

[pld3230-bib-0030] Su, W. , Zhang, M. , Bian, D. , Liu, Z. , Huang, J. , Wang, W. , … Guo, H. (2019). Phenotyping of corn plants using unmanned aerial vehicle (UAV) images. Remote Sensing, 11(17), 2021 10.3390/rs11172021

[pld3230-bib-0031] Tetio‐Kagho, F. , & Gardner, F. P. (1988). Responses of maize to plant population density. I Canopy development, light relationships and vegetative growth. Agronomy Journal, 80, 930–935.

[pld3230-bib-0032] Varela, S. , Assefa, Y. , Prasad, P. V. , Peralta, N. R. , Griffin, T. W. , Sharda, A. , … Ciampitti, I. A. (2017). Spatio‐temporal evaluation of plant height in corn via unmanned aerial systems. Journal of Applied Remote Sensing, 11(3), 1 10.1117/1.JRS.11.036013

[pld3230-bib-0033] Watanabe, K. , Wei, J. , Arai, K. , Takanashi, H. , Kajiya‐Kanegae, H. , Kobayashi, M. , … Iwata, H. (2017). High‐throughput phenotyping of sorghum plant height using an unmanned aerial vehicle and its application to genomic prediction modeling. Frontiers in Plant Science, 8, 421 10.3389/fpls.2017.00421 28400784 PMC5368247

[pld3230-bib-0034] Xavier, A. , Hall, B. J. , Hearst, A. A. , Cherkauer, K. A. , & Rainey, K. M. (2017). Genetic architecture of phenomic‐enabled canopy coverage in Glycine max. Genetics, 206(2), 1081–1089. 10.1534/genetics.116.198713 28363978 PMC5499164

[pld3230-bib-0035] Yang, G. , Liu, J. , Zhao, C. , Li, Z. , Huang, Y. , Yu, H. , … Yang, H. (2017). Unmanned aerial vehicle remote sensing for field‐based crop phenotyping: Current status and perspectives. Frontiers in Plant Science, 8, 1111 10.3389/fpls.2017.01111 28713402 PMC5492853

[pld3230-bib-0036] Yin, X. , Jaja, N. , Mcclure, M. A. , & Hayes, R. M. (2011). Comparison of models in assessing relationship of corn yield with plant height measured during early‐ to mid‐season. Journal of Agricultural Science, 3(3), 14–24. 10.5539/jas.v3n3p14

[pld3230-bib-0037] Yin, X. , Mcclure, M. A. , Jaja, N. , Tyler, D. D. , & Hayes, R. M. (2011). In‐season prediction of corn yield using plant height under major production systems. Agronomy Journal, 103, 923–929. 10.2134/agronj2010.0450

