# Supplementary material for: UAV‐based imaging platform for monitoring maize growth throughout development
Source: Plant Direct. 2020 Jun 8;4(6):e00230. doi: 10.1002/pld3.230 (PMC7278367; doi:10.1002/pld3.230)
Supplement: Supplementary file 1 — Data S1 [file PLD3-4-e00230-s001.pdf]

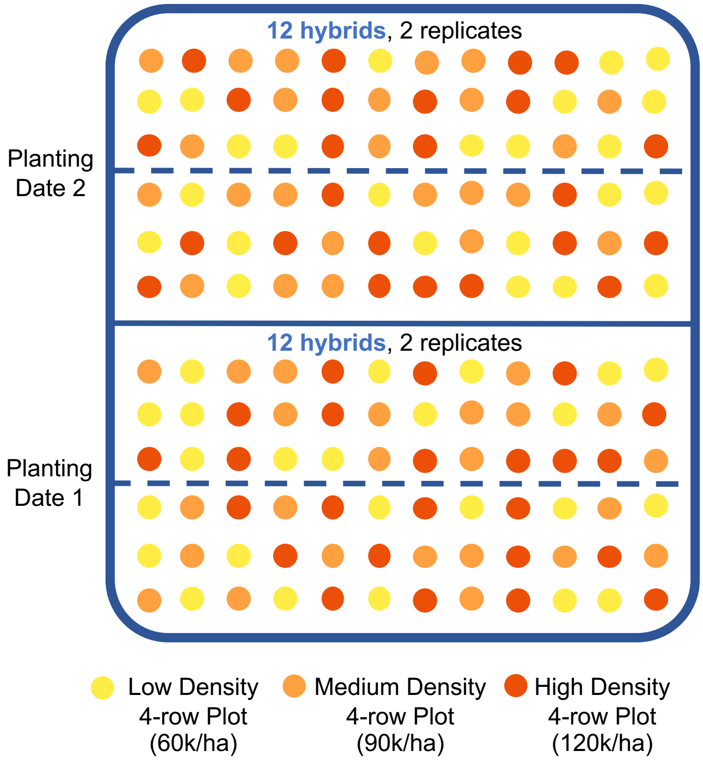

Figure S1. Field experimental layout for 2018 biological material.

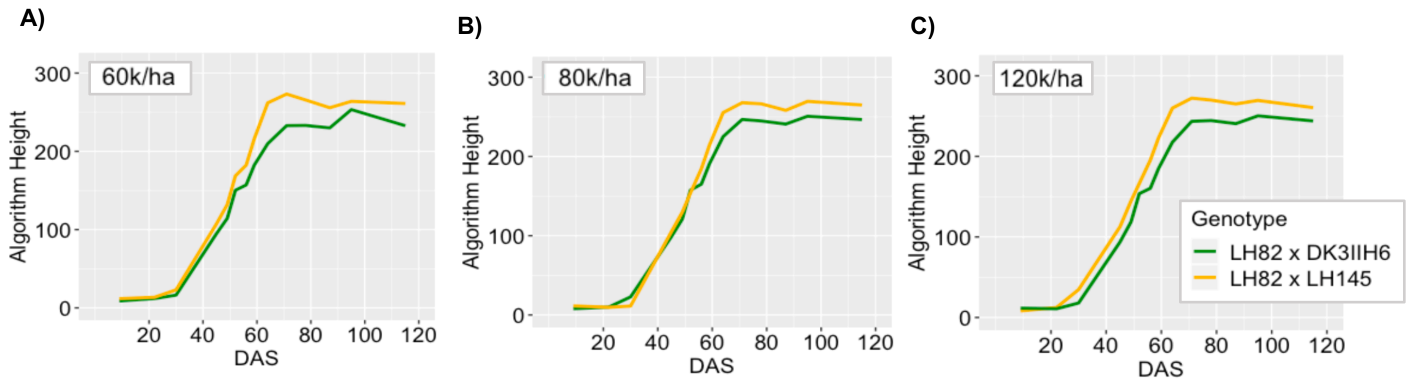

Figure S2. Height through time for various genotypes and treatments. A-C) Height through time as measured by the UAV for plots of two different genotypes (LH82 x DK3IIH6 and LH82 x LH145) in the late planting date treatment and the low (A), medium (B) and high (C) planting densities.

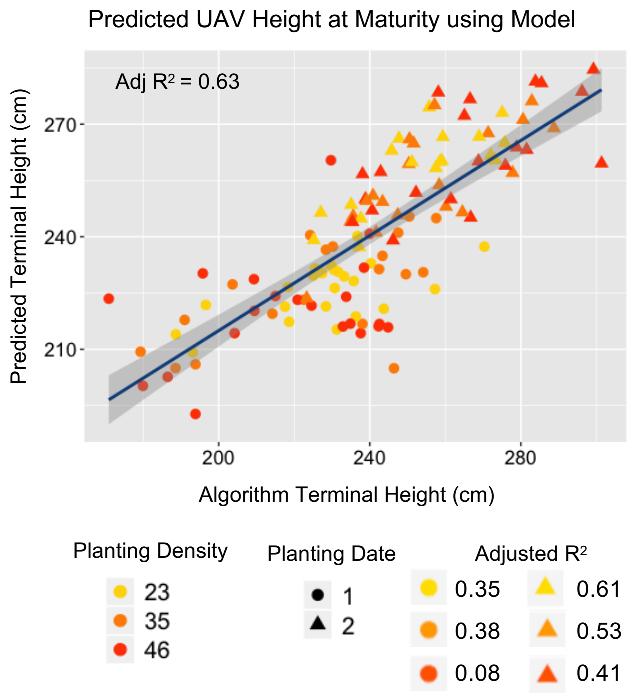

Figure S3. Correlation of predicted terminal height on the test dataset. Predictions were done utilizing a linear regression model derived from the training dataset based on slope values of selected timepoints and the UAV derived height values at maturity.

	PH-R Replicates for Individual Plants			Plot Mean PH-UAV to PH-R(2) for Plot Subset			Plot Mean PH-R(2) Replicates for Plot Subset		
	**Adj R2**	**RMSE**	**NRMSE**	**Adj R2**	**RMSE**	**NRMSE**	**Adj R2**	**RMSE**	**NRMSE**
6/13/18	0.45	4.50	0.12	0.05	12.29	0.34	0.64	2.52	0.07
7/17/18	0.97	7.75	0.03	0.47	27.36	0.11	0.88	4.00	0.02
8/9/18	0.90	7.18	0.03	0.72	27.19	0.11	0.98	3.00	0.01

Table S1. Adjusted r-square values and root mean square error, and normalized root mean square error by mean for the linear correlation of various PH measurements. From left to right: replicated hand measurements obtained for 2 plants across 12 plots, means derived from the replicated hand measurements obtained for 2 plants of the same 12 plots, algorithm-derived plot mean height values to the respective hand-measured plot mean height of the same 12 plots, and model-derived plot mean height values compared to the respective hand-measured plot mean height of the same 12 plots.

	Degrees of Freedom	Sum of Squares	Mean Sum of Squares	Percent of total variation
Planting Date	1	46485	46485	43.37***
Genotype	11	11316	1029	10.55**
Density	2	148	74	N.S.
Planting Date:Genotype	11	4582	417	N.S.
Density:Genotype	22	6218	283	N.S.
Planting Date:Density	2	3078	1539	2.87*
Residuals	90	35365	393	N.S.
* significant at *p* =0.05; ** significant at *p* =0.01; *** significant at *p* =0.001; N.S., not significant.				

Table S2. Analysis of variance of UAV-derived terminal plant height for 12 hybrids planted in a randomized block design with density and planting date treatments.
